# The transformation of Jordan’s healthcare system in an area of conflict

**DOI:** 10.1186/s12913-024-11467-1

**Published:** 2024-09-06

**Authors:** Ahmad Tamimi, Mousa Al-Abbadi, Iskandar Tamimi, Malik Juweid, Muayyad Ahmad, Faleh Tamimi

**Affiliations:** 1 Department of Neurosurgery, Faculty of Medicine, University of Jordan, Amman, Jordan; 2 Department of Pathology and Cytopathology, Faculty of Medicine, Jordan University Hospital, University of Jordan, Amman, Jordan; 3 Orthopedic Surgery Department; Regional University Hospital of Malaga, Malaga University, Malaga, Spain; 4 Department of Radiology and Nuclear Medicine, Faculty of Medicine, University Hospital, University of Jordan, Amman, Jordan; 5 Department of Clinical Nursing, Faculty of Nursing, University of Jordan, Amman, Jordan; 6 College of Dental Medicine, Qatar University, Doha, Qatar

**Keywords:** Healthcare system, Health Insurance, Medical Education, Medical tourism, Jordan

## Abstract

**Background:**

The Jordanian healthcare system has evolved over the past decades expanding its services, technological, and educational resources. A comprehensive view of this system is lacking. The objective of this report is to describe the structure of the Jordanian healthcare system, the challenges facing it, and the current and recommended health policies.

**Materials and methods:**

This study reviewed the current status of the Jordanian healthcare system. The following parameters were analyzed: health indicators, infrastructure, human resources, insurance system, pharmaceutical expense, health education system, and medical tourism. Data were collected from various relevant official institutions and related published literature.

**Results:**

Jordan has a young population with a median age of 23.8 years. Life expectancy is 78.8 years for females and 77.0 years for males. The Jordanian healthcare system is divided into three major categories: (1) Governmental Insurance (i.e., the Ministry of Health (MOH), the Royal Medical Services (RMS) and semi-governmental insurance); (2) Private Insurance; and (3) Refugee Insurance, including the United Nations Relief and Works Agency for Palestine Refugees in the Near East (UNRWA) and the United Nations High Commissioner for Refugees (NHUR). The Governmental Insurance covers 64.30% of the total population. Health expenditure is 6.37% of the gross domestic product (GDP). Pharmaceutical expenses make up 26.6% of the total national healthcare budget. Human resource assessment shows a high ratio of medical staff per 10.000 inhabitants, especially concerning physicians (31.7), dentists (7.9), and pharmacists (15.1). However, the ratio of nursing staff per 10.000 inhabitants is considered low (37.5). The Hospital bed/1000 population ratio is also relatively low (1.4). Healthcare accreditation is implemented through the Joint Commission International (JCI) accreditation which was achieved by 7 hospitals and by the National Health Care Accreditation Certificate (HCAC) achieved by 17 hospitals and 42 primary healthcare centers. Postgraduate medical education covers almost all medical and surgical fields. Medical tourism is currently well-established.

**Conclusions:**

Assessment of the Jordanian healthcare system shows high ratios of physicians, dentists, and pharmacists but a low ratio of nursing staff per 10.000 inhabitants. The hospital bed/1000 population ratio is also relatively low. Pharmaceutical expenses are significantly high and medical tourism is well-developed.

## Introduction

Jordan is a Middle Eastern Arabic country covering an area of 89,342 km with a total population of 11.517.887 inhabitants [1]. Jordan is considered a low-middle-income country with a gross domestic product (GDP) of 48.65B USD/year [1] and a GDP per Capita of 4.255.00 USD/year [2]. In addition, Jordan hosts the second-highest share of refugees per capita worldwide [3].

The Jordanian healthcare system has evolved over the past decades transforming into a generally well-developed service despite being in an area of constant conflicts and volatility, particularly in neighboring countries, such as Palestine, Syria, and Iraq.

During the last three decades, the health status of the Jordanian population has significantly improved. For example, since 1990, life expectancy has increased from 69.85 to currently 77 years for males and 78.8 years for females [4]. Infant mortality has decreased from 32.0 to 12.8 per 1,000 live births in 2024(6). In addition, Jordan has become a popular destination for medical tourism in the region.

The country has one of the most modern health systems in the region and the total health expenditure was estimated at 6.73% of the GDP [5]. The national policies in Jordan have been oriented towards expanding the health care umbrella to cover all Jordanian citizens.

**Fig. 1:**
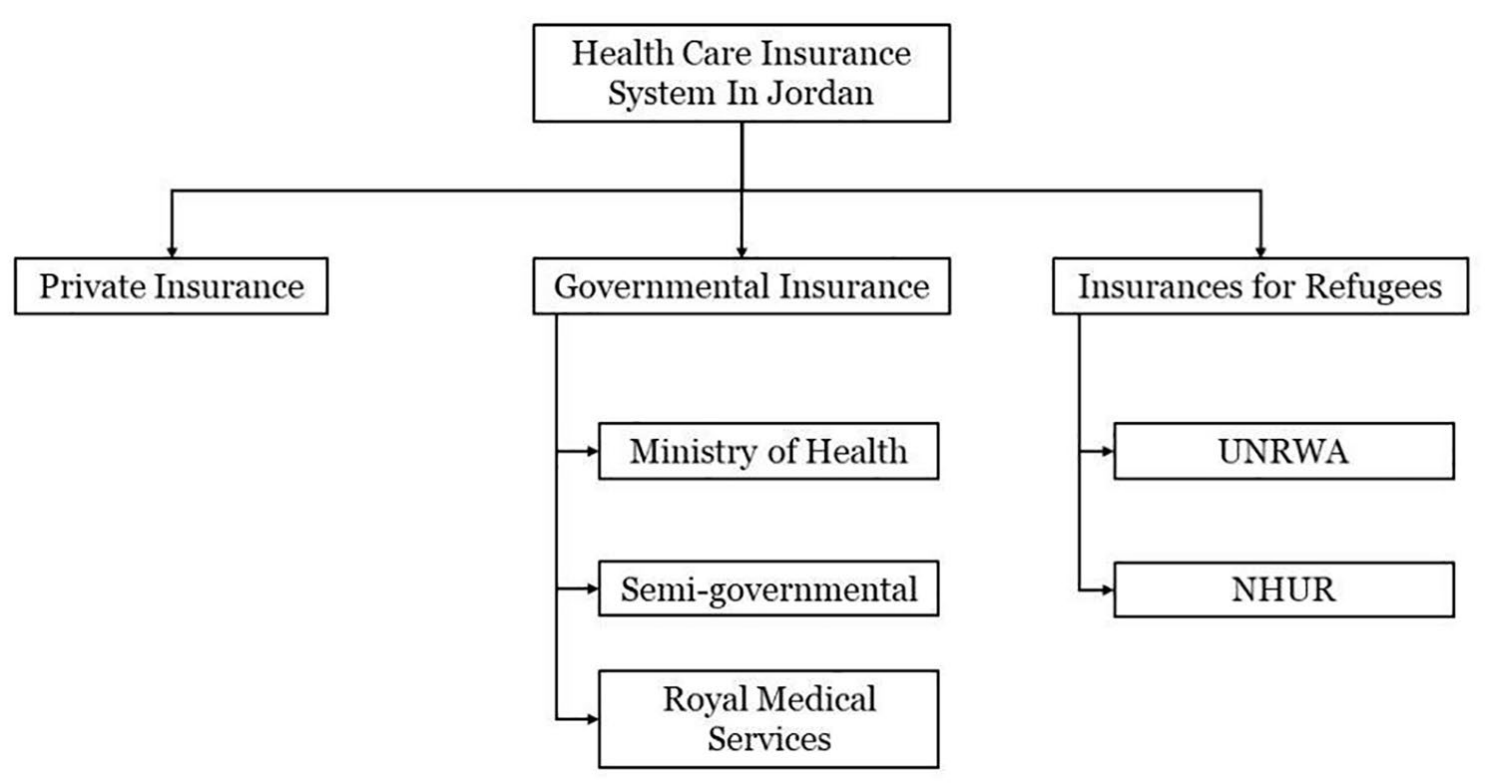
Structure of the Healthcare System in Jordan

The Jordanian healthcare system provides wide public healthcare services with a relatively low budget and is divided into a public system, private sector, and refugee insurance system. The public system is subdivided into the Ministry of Health (MOH), the Royal Medical Services (RMS), and semi-governmental insurance, such as the Jordan University Hospital and King Abdallah the First Hospital. The refugee insurance system is subdivided into the United Nations Relief and Works Agency for Palestine Refugees in the Near East (UNRWA) and the United Nations High Commissioner for Refugees (NHUR) (Fig. 1). The governmental and semi-governmental insurances cover approximately 64.3% of the Jordanian population [6, 7].

A comprehensive assessment of the Jordanian healthcare system is currently lacking.

In this article, we describe the structure of the Jordanian healthcare system, the challenges facing it, current and recommended health policies.

## Methods

In this study, we analyzed the following parameters of the healthcare system: infrastructure (hospitals, number of beds, primary healthcare centers), human resources (physicians, dentists, pharmacists, nurses), insurance system (variety of insurance systems), pharmaceutical expenses, healthcare educational teaching system (undergraduate and postgraduate) and medical tourism.

Data were obtained from various relevant official resources and institutions, such as the MOH, Jordan High Council for Health (JHCH), National Statistics Department (NSD), Ministry of Higher Education (MOHE), Jordanian Universities, Royal Court (RC), National Department of Civil Status (NDCS), health care teaching, World Health Organization (WHO), the UNRWA and World Bank. We also utilized up-to-date related published data. We excluded any data that was not officially published in PubMed nor reported by the official Jordanian organizations in order to avoid inaccurate data. This research project was sponsored by the University of Jordan (R.1511/2023/19). It did not require approval by our institutional ethical committee.

## Results

### Jordanian Administration System

Jordan is a constitutional monarchy in which the king is the head of state. He exercises his executive power through the Prime Minister and the Council of Ministers. The House of Representatives is democratically elected by Jordanians and the members of the Senate are appointed by the king. In addition, there is an independent judiciary system. Jordan is divided into 12 Governorates subdivided into 54 municipalities [8].

The Jordanian economy is classified as an emerging market with a mixed economic system, including a private economy combined with centralized economic planning and governmental regulation [9].

### Demography and health indicators

According to the national official statistics from 2023, the total population of Jordan was 11.517.887 [8.514069 (73.92%) Jordanians and 3.003818 (26.08%) non-Jordanians], including 5.344299 (46.4%) females and 6.173588 (53.6%) males [10].

The age range of 0–14 years constitutes 30.7% of the population, and individuals between 15 and 65 years and over 65 years constitute 65.9% and 3.7% of the population, respectively (Table 1) [9]. The median population age is 24 years [11].

**Table 1:** Demographic and health indicators data in Jordan

Indicators	Year 2022
Population	11.517.887
Male	53.6%
Female	46.4%
Median Age (years)	24.0
0–14	30.4%
15–64	65.9%
≥ 65	3.7%
Fertility rate (per female)	2.22
Infantile Mortality ≥ 5years Per 1000 live birth	12.8(2024)
Birth/year/1000 population	21.1
Life Expectancy (years):	
Male Female	77.0 78.8

The unemployment rate is still above the pre-pandemic levels (22.6%), especially among women (29.4%) and the young (46.1% among those under 25 years). The female labor force is particularly small, being one of the smallest in the world [12]. Life expectancy for females and males was 78.8 and 77.0 years, respectively. The infantile mortality below the age of 5 years was 12.8 /1000 live births whilst the fertility rate was 2.22 children per woman [9, 13]. (Table 1).

**Fig. 2:**
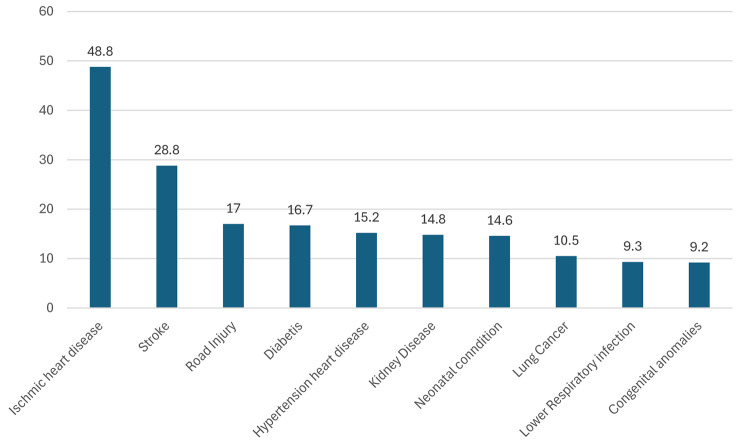
Top causes of death/100.000 population in Jordan(WHO)

Disease patterns have also changed in Jordan, alongside the economic development, from predominantly acute to chronic diseases. Today the leading causes of death per 100.000 inhabitants are ischemic heart disease (48.08), cerebrovascular accidents (28.8), road traffic accidents (RTA) (17.0), diabetes mellitus (16.7), and lung cancer (10.84) (Fig. 2) [14]. Two important burdens on the health system are smoking and obesity. The prevalence of smoking among Jordanians is very high, with a prevalence of smokers under the age of 45 years of 85% [15] with a male-to-female ratio is 53.6%, to 46.4%. Obesity, a global public health problem in terms of morbidity and mortality is prevalent in the country. According to the waist-to-height ratio (WHtR) measurement, 44.2% of Jordanian men and 47.8% of Jordanian women are considered obese [16].

### Health Insurance System

Overall, 76.80% of the Jordanian population is covered by some healthcare insurance system (Table 2). Among non-Jordanians who are not considered refugees, this figure is 25.3% [17]. The Jordanian health insurance system can be divided into three different categories (Table 2):

**Table 2:** Health Insurance distributions

Entity	Jordanians (8.514.069)	Jordanian Insured (%)
MOH	3,490000	41.0
RMS	1,985121	23.31
UHs	110,000	1.3
*Total public insurance*	*5*,*585121*	*65.6*
UNRWA	-	6.8
NHUR	-	NA
Private Insurance	NA	12.5
*Total insured*	*6*,*538678*	*76.8*

Abbreviations: Ministry of Health, MOH; Royal Medical Services, RMS; University Hospitals, UHs; United Nations Relief and Works Agency for Palestine Refugees in the Near East, UNRWA; United Nations High Commissioner for Refugees, NHUR; Non-available, NA

1. Governmental insurance, which is subdivided into the MOH, the RMS, and semi-governmental insurance. A total of 65.59% of Jordanian citizens are covered by governmental insurance. The MOH insurance covers governmental employees and their dependent family members (i.e.,40.99%) [6]. In addition, all children less than 6 years old and citizens aged above 60 years are freely covered by the MOH. The RMS covers military members and their dependents (i.e., 23.31%) [7], and the semi-governmental services cover university employees, their dependents as well as university students (i.e., 1.29%) [17].
2. Private insurance: it includes private institutions that are responsible for the health insurance of their personnel and independent individuals who purchase private insurance (i.e., 12.5%) [18].
3. Refugee insurance: It includes mainly primary healthcare. The UNRWA is responsible for the healthcare of Palestinian refugees (i.e., 6.8%) [17] while NHUR covers Syrian refugees (Table 2).

Primary healthcare clinics in Jordan provide quick access to medical care, vaccination schedules, maternity, childcare, and chronic disease management services. They operate in urban as well as rural areas and range in size from small individual clinics to comprehensive multi-clinic centers, depending on the area and its population.

### Health Expenditure

- The national expenditure on public health arises from services provided by the MOH, RMC, and semi-governmental agencies and represents 6.4% of the GDP. This expenditure covers 65.6% of the Jordanian population (Table 3). The costs of governmental and private sector services constitute 64.3%, and 31.75% of the total healthcare expenditure, respectively [18].
- The in-hospital services represent 69.55% of the national health expenditure, whereas 21% is spent on primary care, 7.17% on administrative activities, 1.62% on training, and less than 1% on miscellaneous activities [19] (Table 4).
- The MOH insurance also covers the dependent family members of contributing individuals. Affiliated subjects pay 3% of their gross salaries per month, with an upper limit of 30 JD/month. Coverage includes all potential health conditions and their respective treatments [20] (Table 4).
- In 2022, the average cost per patient admission was 782.3 JD regardless of the length of hospitalization. Moreover, the mean inpatient day cost was 236.6 JD, and the bed day cost (i.e. without additional medical procedures) was 172.9 JD.
- The average operation cost was 449.6 JD and the per emergency room visit cost was 31.8 JD [21].
- The outpatient clinic expenditures (out of pocket) were 28.8 JD and 6.8 JD at private and public facilities, respectively [22] (Table 4).
- The pharmaceutical and disposable expenditures accounted for 15.15% of the total health expenditure budget of the MOH whilst 26.6% of the total health expenditure was attributed to just the pharmaceutical expenses [18]. However, medication fees for the insured are symbolic, ranging from 0.25 to 10 JD per drug. Other types of insurance have followed similar payment criteria [6, 7].

**Table 3:** Healthcare expenditure ratio and indicators

Data	Health expenditure (%)
Gross Domestic Product	6.7
Expenditure in public health	64.3
Expenditure in Private health	31.6
Miscellaneous	4.0
**Expenditure/type of service**	
Curative care	69.4
Primary care	21.0
Administrative	7.17
Training	1.6
Miscellaneous	1.0
**Pharmaceutical expenses**	
Pharmaceutical	26.6

**Table 4:** Hospital and outpatient costs (JD)

Hospital costs (JD)	MOH	RMS	UHs	Average
Cost per admission	557	914	740	782.3
Cost per inpatient day	175	277	230	236.6
Cost per bed day	119	207	162	172.9
Cost per operation	402	548	399	449.6
Cost per emergency room visit	22	26	47	31.8
**Outpatient cost (JD)**				
Cost per outpatient visit	24	71	62	58.4
Out of pocket: Cost per outpatient visit in public service	NA	NA	NA	6.8
Out of pocket: Cost per outpatient visit in private service	NA	NA	NA	28.8

Abbreviations: Jordanian Dinar, JD; Ministry of Health, MOH; Royal Medical Services, RMS; University Hospitals, UHs; Non-available, NA

Syrian refugees place an important burden on the healthcare system in Jordan. The cost of healthcare for Syrians in Jordan is 271 million JD/year [23]. The Government’s policy on Syrian refugees’ access to healthcare services has changed over the last 10 years. At the beginning of the Syrian crisis, the government granted free of charge access to the Jordanian public health system. However, by 2015 Syrian refugees followed the same insurance criteria as Jordanians. In 2018, the policy was reversed with Syrian refugees having to pay 80% of the established fees for foreigners in Jordan at the MOH facilities. However, Syrian refugees were still exempt from fees for maternity and childhood services [18].

### Hospital beds and primary health care

- Tertiary health care in Jordan includes 120 hospitals [i.e., 31 MOH hospitals with 5884 beds, 17 RMS hospitals with 3350 beds, 2 university hospitals with 1236 beds and 70 private hospitals with a total of 5529 beds]. Accordingly, the total number of hospital beds in Jordan is 15.999 (1.45/1000 population). (Table 5)
- There are a total of 832 primary healthcare clinics and 440 dental clinics [9].

**Table 5:** Hospital bed distribution and occupancy rate

Institutions	Hospital Number	Bed Number	Percent (%)	Occupancy rate (%)
MOH	31	5884	35.2	64.7
RMS	17	3350	20.17	66.1
UHs	2	1236	8.1	68
Private Hospitals	70	5529	36.0	41.4
Total	120	15,999	100.0	58.6

Abbreviations: Ministry of Health, MOH; Royal Medical Services, RMS; University Hospitals, UHs

### Human resources

The ratio of healthcare professionals in Jordan per 10.0000 population is 31.7 for physicians [4], 9 for dentists, 15.1 for pharmacists, and 37.5 for nursing staff (Table 6) [9].

**Table 6:** Health care and human resources: comparison between 12 countries (per 10000 population)

Professionals	Jordan	USA	Japan	UK	Germany	Spain	Turkey	Greece	Egypt	Israel	Tunis	Lebanon
Physicians	31.7	35.5	26.1	32	45	45	20	63	7.1	37	26	26
Dentists	7.9	6	8.3	5.2	8.5	8.4	4.1	13	1.9	8.9	2.9	12
Pharmacist	15.1	11	20	8.5	6.7	13	4.2	11	4.3	8.4	2.2	15
Nurses	37.5	125	125	91.7	123	63.1	34	37	18.3	56.3	24.3	19.3

### Accreditation and healthcare

During the last decade, there has been an increasing awareness of the importance of accreditation in healthcare activities. Accordingly, 7 hospitals were accredited by the Joint Commission International (JCI) [i.e., 2 university hospitals, and 5 private hospitals] as well as a private laboratory [24].

The National Health Care Accreditation Council (HCAC) also accredited 17 hospitals from the public, private, and academic sectors and 42 public primary health care centers. In addition, it also certified two diagnostic imaging centers [25]. This has led to a significant improvement in the overall quality of the Jordanian healthcare system.

### Medical education

#### Undergraduate medical education

Jordan’s undergraduate medical education started in the 1970s with the establishment of the first medical school at the University of Jordan [24]. There is a relatively large number of medical and dentistry schools in the country (i.e., 8 and 6, respectively).

Admission to medical schools depends exclusively on grades obtained in the Jordanian General Secondary Certificate exam (JGSC) or equivalent. The students who meet the minimum requirements in the science JGSC track (i.e., a score of 85%) or equivalent are eligible to apply for medical schools in Jordan. No additional cognitive or non-cognitive tests are required for this purpose.

The admission system to Jordanian medical schools includes multiple tracks [26, 27]: (a) The open National Unified Admission (NUA) track; (b) a track for underprivileged students; (c) for the children of university employees (UES); (d) the parallel track (i.e. this is a track for students with lower scores that are required to pay higher registration university fees) and (e) The international track for foreign students.

The curriculum is divided into an initial 3-year pre-clinical or basic medical science stage (integrated system) followed by 3 clinical years (traditional). At the end of the sixth year, students undergo a written theoretical assessment as well as a supervised clinical assessment. The students who successfully pass the exams are awarded a medical doctor degree provided they also complete a research project, which is evaluated by a faculty committee during the last semester of the program [26, 28].

There are currently approximately 20,609 medical students at Jordanian universities with a similar number studying abroad [29]. However, there is a relatively low number of nursing students and a high number of dentistry and pharmacy students (Table 7). Approximately, 3,000 students graduate from Jordanian medical schools annually, whilst another 1000 students graduate from countries abroad [30].

**Table 7:** Students registered in the health schools in the academic year 2022/2023

Undergraduate	Jordanians	Non-Jordanians (%)	Total
Medical schools	16,749	3899(18.9)	20,648
Dentistry School	3351	837(19.20)	4188
Nursing School	9335	1062(10.2)	10,397
Pharmacy School	9737	4220(30.2)	13,957

#### Postgraduate medical education

A partial postgraduate specialty medical training program (i.e. 2–3 years of training) was established in 1968 at the RMS and some MOH hospitals. This resulted in residents having to complete their training in centers abroad to obtain their official specialty degree. In 1982, the Jordan Medical Council (JMC) was established to control the practice of medical specialties in the country, and the first complete residency programs were established at Jordan University Hospital. This was followed by similar programs at other MOH and RMS hospitals as well as the Jordan University of Science and Technology Hospital and, more recently in some private hospitals and the King Hussein Cancer Center [31]. The approximate number of the offered medical training positions is 1200 posts/year nationwide.

Postgraduate medical education in Jordan is oriented towards the acquisition of specialty degrees in all the major clinical specialties and some subspecialties. The admission criteria to postgraduate medical and surgical training programs are based on a theoretical exam and an interview, both performed by each training center and accredited by the JMC. The duration of the training period varies between specialties, ranging between 4 and 6 years, and the trainees are evaluated each year [26]. To qualify as specialists, the trainees must pass the JMC competency assessment at the end of the program. This assessment is supervised by a select committee of senior qualified specialists and is divided into two parts: (a) Part 1; a multiple-choice questions (MCQ) exam that includes basic medical and clinical sciences related to the specialty and (b) Part 2 comprised of a comprehensive assessment that includes specialty-specific MCQs, oral and clinical exams. After passing these final exams, the residents are qualified to practice as specialists throughout the country. Specialists trained in other countries must also pass these exams to be eligible to practice in Jordan. In 2023, new legislation exempted from the JMC exams postgraduates from countries abroad who hold a foreign board certificate and have 3 years of experience as specialists in the country they graduated from [31].

Regarding the postgraduate academic track, there are currently variable tracts of master’s degree programs in basic medical sciences and only one PhD program in medical statistics.

### Medical health tourism

The tourist industry’s contribution to the global GDP reached 10.3% in 2019 and was responsible for the creation of one out of every four new employments worldwide [32]. In Jordan, there are approximately 55,000 jobs directly related to tourism with an additional 125,000 jobs indirectly related [28]. The state of the tourism sector is widely regarded as ¨below potential¨, especially given the country’s rich history, ancient ruins, Mediterranean climate, and diverse geography.

Medical tourism in Jordan has progressively become more relevant as a result of the good reputation of the Jordanian healthcare system regionally. Medical tourism constitutes 3.5% of the country’s GDP [33]. This sector, which has a high added value and creates employment in many areas is supported by the government with investments and incentives [32, 34].

The competitive edge of medical tourism arises from its cost-effective health services, its renowned healthcare reputation, and the diversity of its touristic attractions. In addition, the majority of the medical tourists who visit Jordan come from other Arab countries, as Jordan offers a similar culture and no language barrier.

## Discussion

The Kingdom of Jordan is a low to middle-income country, with a population having a median age of 24 years [9], which is below the average world age (i.e. 30.5 years) [11]. However, with the continuous increase in life expectancy and decrease in the fertility rate [8], the average age of the Jordanian population is expected to increase in the coming years.

Jordan is in an area of constant conflicts and volatility, particularly in neighboring countries, such as Palestine, Syria, and Iraq, affecting its economy and constituting a significant burden on its healthcare system. The country has a high percentage of immigrants (i.e. 33.89%) compared with other countries that are traditional receivers of immigrants (i.e. Australia 30.14%; Canada 21.33%; Germany 18.81%; the USA 15.28% and the UK 13.79%) [35]. Syrian refugees have placed significant challenges on the Jordanian healthcare system, especially because of the high prevalence of women and children, wounded patients, individuals suffering from mental health conditions, and elderly patients. These vulnerable groups require a wide range of expensive services [34]. In addition to affecting the healthcare system, the high number of immigrants and refugees has a significant impact on the country´s infrastructure, security, social structure, financial sector, and education.

The kingdom has a high unemployment rate (i.e.,23.5%), that is particularly high among the female population [36]. Accordingly, only 14.7% of women within the working age contribute to the country’s workforce [36] compared with 39.49% worldwide [37]. Nevertheless, there is a high percentage of female undergraduate students in Jordan (i.e. 55%) and this number increases to 65% among healthcare students (Table 7) [8]. However, many of these female students decide not to work after concluding their studies attributed to multiple factors. We, therefore, believe that there is a need to readjust the current legislation to encourage women to participate in the national labor market.

On the other hand, the different causes of death and morbidity also have an additional burden on the health care system. The leading causes of death are similar to those in lower-middle-income countries. However, the incidence of road traffic accidents (RTA) is particularly high (i.e. 18/100,000 inhabitants) when compared with lower-middle-income and other countries (i.e. the UK, Spain, Germany, and Australia where the death ratio due to RTA is less than 5/100.000 population) [11, 38]. The morbidities and disabilities caused by RTAs, constitute an additional burden on the Jordanian healthcare system with an estimated cost of RTAs of 324 million JD/year, according to the Jordan Traffic Institute [39].

The smoking rate in Jordan is one of the highest in the world, with a prevalence of 70.2% among adults [40]. The male/female ratio is 53.6% /46.4% [9]. Moreover, the prevalence of smokers below the age of 45 is 85% [15], making it the highest among eastern Mediterranean countries and the second highest worldwide after Indonesia [31, 40]. Smoking is associated either directly or indirectly with approximately one in every 8 deaths in the country. It also has an estimated cost of $2.67 billion annually because of healthcare expenditures and loss of productivity [41]. Therefore, stricter implementation of the law by the different national institutions should be a national priority to reduce the burden of smoking on society.

Obesity is another burden on the economy and healthcare system in Jordan due to associated morbidity and mortality. According to the WHtR measurement, the prevalence of obesity in the Jordanian population is 44.2% in men and 47.8% in women [14]. The prevalence of obesity is alarmingly high and increasing in many countries in the Eastern Mediterranean region due to changes in food consumption, reduced physical activity, and an increasingly sedentary lifestyle [42, 43]. However, obesity in Jordan is also among the highest in the region and higher than in Western countries, such as the USA and UK (i.e. 34% and 24%, respectively) [44]. Consequently, obesity in Jordan results in an estimated cost of 650 M JD annually [45].

The number of hospital beds in Jordan (i.e. 4.4/1000 inhabitants) is relatively low compared with other countries (i.e., 12.65/1000 inhabitants in South Korea) [46]. Moreover, the bed occupancy rate is also low at 58% compared to the Organization for Economic Co-operation and Development (OECD)countries (i.e., 75%). Therefore, there is a need to increase the number of hospital beds and bed occupation efficiency [47], which requires further reinvestment in the infrastructure and a better management of resources.

Jordan has a high number of physicians, dentists, and pharmacists per 10.000 inhabitants [19] and a relatively low number of nurses. These figures require future adjustments to adapt the number of health professionals to the demands of the labor market. Moreover, the number of unemployed physicians is currently approximately 2500 (unpublished data from the records of the Jordan Medical Association).

Public health insurances cover 64.30% of the Jordanian population [17]. However, the government is considering a potential expansion of the public insurance system to include all Jordanians. This step would require an important additional investment increasing the health care expenditure to 9% of the GDP. Nevertheless, the size of the public healthcare insurance is one of the best in the region (e.g., in Egypt, the public health insurance covers 60% of the population) [48] but it is still limited compared with other countries, such as Turkey (90%) [49] and Spain (99%) [50].

The total health expenditure in Jordan was estimated to be 3.17 billion USD (i.e., 6.37% of the GDP), which is similar [20, 21] to other countries such as Greece (8.6%), Israel (7.4%) and Turkey (4.3%) [50]. Expenditure on pharmaceutical products is relatively high in Jordan reaching up to 25.9% of the total health expenditure [22]. This number is higher than the majority of the OCED countries and similar to Greece (27%) and Romania (23.9%) [51].

Global Healthcare Accreditation (GHA) is one of the major players in the global medical travel industry, which identifies and compares medical travel programs and destinations against global standards and international best practices. There has been an early awareness of the importance of the quality of the health system in Jordan. The country involved national and international organizations, to achieve quality certificates, such as those from JCI, JCI-academic centers, and Health Care Accreditation Council (HCAC) [25]. This awareness has had a positive impact on medical tourism, making Jordan a regional leader in this sector. As mentioned above, the number of healthcare graduates should be adjusted to the requirements of the labor market [9]. Moreover, postgraduate medical education has a limited number of training residency programs and infrastructure; and there should be a close supervision of the quality of training programs, by JMC and the Jordan Medical Association (JMA).

Finally, the medical tourism sector in Jordan is highly competitive compared to other countries in the region [34], not just for the competitive costs of treatment but also for the additional services that support health tourism in general.

### Strengths of the study

This is a comprehensive updated study of the healthcare system in Jordan. It includes information on the national health system, infrastructure, human resources, medical education, and health tourism. It also includes updated figures, which can be valuable for experts, stakeholders, and healthcare researchers.

### The weak points

Due to the insufficient data in PubMed, we had to use information from different national institutions and annual reports. Moreover, there was some variation in several estimates depending on the sources (i.e. governmental reports or international organizations).

## Conclusion and recommendations for future research and policy actions

The Jordanian health system is divided into public and private sectors and United Nations refugee services. The healthcare infrastructure needs a larger number of hospitals and a more efficient administration regarding bed occupancy and pharmaceutical expenses. Moreover, Jordan needs the effective support of the international community to cover the health needs of the Syrian and Palestinian refugees. The country also needs to address significant social health problems, such as the high number of road traffic accidents, the prevalence of smoking, and obesity. Moreover, the number of human resources should be altered to increase the number of nursing staff and control the number of graduating physicians and pharmacists. More attention should be paid to postgraduate medical education, including the academic track of PhD programs, especially in our medical schools. Finally, medical tourism should be further developed as the country has multiple assets making it an attractive destination.

## Data Availability

The data are available in the annual reports of the different institutions (MOH, RMS, (JHCH), (NSD), (MOHE), (NDCS), (WHO), the UNRWA, and World Bank).

## Abbreviations

GHA: Global Healthcare Accreditation
GPD: Gross Domestic Products
HCAC: Health Care Accreditation Certificate
JCI: Joint Commission International
JD: Jordan Dinar (1 dollar = 0.71JD)
JGSC: General Secondary Certificate exam
JHCH: Jordan High Council for Health
JMA: Jordan Medical Association
JMC: Jordan Medical Council
MCQ: Multiple-Choice Questions
MOH: Ministry of Health
RMS: Royal Medical Services
UNRWA: United Nations Relief and Works Agency
NHUR: United Nations High Commissioner for Refugees
NSD: National Statistics Department
M: Million
MOHE: Ministry of Higher Education
NDCS: National Department of Civil Status
NUA: National Unified Admission
OECD: Organization for Economic Co-operation and Development
PhD: Philosophy Doctor
RTA: Road Traffic Accidents
RC: Royal Court
UES: University Employees
UK: United Kingdom
USA: United States of America
USD: United States Dolar
WHO: World Health Organization
WHtR: Waist-to-height ratio

## References

[1] World Bank Open. Data [Internet]. [cited 2024 Mar 3]. World Bank Open Data. https://data.worldbank.org

[2] Jordan GDP. Annual Growth Rate [Internet]. [cited 2024 Mar 3]. https://tradingeconomics.com/jordan/gdp-growth-annual

[3] The Global Compact on Refugees | UNHCR [Internet]. [cited 2024 Mar 3]. The Jordan River Foundation helps bring job opportunities to refugees. https://globalcompactrefugees.org/news-stories/jordan-river-foundation-helps-bring-job-opportunities-refugees

[4] Jordan - Health Indicators. - Humanitarian Data Exchange [Internet]. [cited 2024 Mar 3]. https://data.humdata.org/dataset/d14b4d2b-65fa-4e95-bdfe-e1e88006b124

[5] Jordan JO. Current Health Expenditure: % of GDP | Economic Indicators | CEIC [Internet]. [cited 2024 Mar 3]. https://www.ceicdata.com/en/jordan/health-statistics/jo-current-health-expenditure--of-gdp

[6] Civilian Health Insurance in. Jordan [Internet]. [cited 2024 Mar 3]. https://moh.gov.jo/Ar/NewsDetails/

[7] Military health insurance in Jordan [Internet]. [cited 2024 Mar 3]. https://jrms.jaf.mil.jo/Files/4e8f2ae6-ce45-4cde-9712-3cd18acf79ec.pdf

[8] Jordan. In: Wikipedia [Internet]. 2024 [cited 2024 Mar 3]. https://en.wikipedia.org/w/index.php?title=Jordan&oldid=1211245951

[9] Department of Statistics [Internet]. [cited 2024 Mar 3]. Jordan Statistical Yearbook 2022. https://dosweb.dos.gov.jo/product/jordan-statistical-yearbook-2014/

[10] Jordan Population. (2024) - Worldometer [Internet]. [cited 2024 Mar 3]. https://www.worldometers.info/world-population/jordan-population/

[11] World Demographics 2023 (Population, Age, Sex, Trends) - Worldometer [Internet]. [cited 2024 Mar 3]. https://www.worldometers.info/demographics/world-demographics/

[12] World Bank [Internet]. [cited 2024 Mar 3]. Overview. https://www.worldbank.org/en/country/jordan/overview

[13] United nations world prophylactic prospects). Jordan Infant Mortality Rate 1950–2024. www.macrotrends.net. Retrieved 2024-06-24.

[14] Global health estimates. Leading causes of death [Internet]. [cited 2024 Mar 3]. https://www.who.int/data/gho/data/themes/mortality-and-global-health-estimates/ghe-leading-causes-of-death

[15] Alkouri O, Khader Y, Al-Bashaireh AM. Prevalence of cigarettes and Waterpipe Smoking among jordanians, refugees, and migrants in Jordan and its Associated factors: a secondary data analysis. Int J Environ Res Public Health. 2022;20(1):82. 36612400 10.3390/ijerph20010082 PMC9819960

[16] Ajlouni K, Khader Y, Batieha A, Jaddou H, El-Khateeb M. An alarmingly high and increasing prevalence of obesity in Jordan. Epidemiol Health. 2020;42:e2020040. 32512659 10.4178/epih.e2020040 PMC7871146

[17] Health Insurance in Jordan.pdf [Internet]. [cited 2024 Mar 3]. http://www.dos.gov.jo/dos_home_e/main/population/census2015/Health%20Insurance%20in%20Jordan.pdf

[18] Rawabdeh AA, Khassawneh AS. Health Financing Policies in Jordan: The Allocation of Public Expenditures in Global Context. Makara J Health Res [Internet]. 2018 Dec 28 [cited 2024 Mar 3];22(3). https://scholarhub.ui.ac.id/mjhr/vol22/iss3/8/

[19] Jordan National Health Accounts. 2008 Technical Report No.2 [Internet]. [cited 2024 Mar 3]. http://www.hhc.gov.jo/uploadedimages/6a2f97aa-1863-45cf-8436-01cc2ad9b909.pdf

[20] Tareq SM, Jin H, Kitsios E, Khemani P. Jordan Public Expenditure Review and Rationalization: Issues and Reform Options.

[21] Hammad EA, Alabbadi I, Taissir F, Hajjwi M, Obeidat NM, Alefan Q, et al. Hospital unit costs in Jordan: insights from a country facing competing health demands and striving for universal health coverage. Health Econ Rev. 2022;12(1):11. 35124740 10.1186/s13561-022-00356-0 PMC8818182

[22] Hrh MH. Determinants of Health Insurance Coverage and Out-of-pocket Payments for Health Care in Jordan: Secondary Analysis of the 2017-18 JPFHS.

[23] Syrians’ healthcare. cost amounts to JD271m annually | Jordan Times [Internet]. [cited 2024 Mar 3]. https://www.jordantimes.com/news/local/syrians%E2%80%99-healthcare-cost-amounts-jd271m-annually

[24] The Effect of Talent Management on Organizational Effectiveness in Healthcare Sector by Dr. Bader Yousef Obeidat, Haneen Yassin, Ra’ed Masa’deh :: SSRN [Internet]. [cited 2024 Mar 3]. https://papers.ssrn.com/sol3/papers.cfm?abstract_id=3300271

[25] Jordan Healthcare Accreditation Project (JHAP). - URC [Internet]. [cited 2024 Mar 3]. https://www.urc-chs.com/projects/jhap/

[26] Tamimi AF, Tamimi F. Medical education in Jordan. Med Teach. 2010;32(1):36–40. 20095772 10.3109/01421590903196953

[27] Tamimi A, Hassuneh M, Tamimi I, Juweid M, Shibli D, AlMasri B, et al. Admission criteria and academic performance in medical school. BMC Med Educ. 2023;23(1):273. 37085824 10.1186/s12909-023-04251-y PMC10122404

[28] Medical Schools [Internet]. Association of American Medical Colleges. https://www.aamc.org/news-insights/press-releases/us-medical-school-enrollment-surpasses-expansion-goal

[29] Students number at Jordan University public. and private in the academic year 2022/2023 [Internet]. [cited 2024 Mar 3]. https://www.admhec.gov.jo/mjr2017/UnivMajorCapacity.aspx

[30] annual_report_of_jnc-english_. pdf [Internet]. [cited 2024 Mar 3]. https://jnc.gov.jo/ebv4.0/root_storage/en/eb_list_page/annual_report_of_jnc-english_.pdf

[31] Jordan Medical Council. - Law modification in 2022; official government newspaper [Internet]. [cited 2024 Mar 3]. https://pm.gov.jo/Ar/Pages/NewsPaperDetails/5820

[32] Saleh AS, Assaf AG, Ihalanayake R, Lung S. A panel Cointegration Analysis of the impact of tourism on economic growth: evidence from the Middle East Region. Int J Tour Res. 2015;17(3):209–20. 10.1002/jtr.1976

[33] https://www.esc.jo/Homeen.aspx [Internet]. [cited 2024 Mar 3]. https://www.esc.jo/Homeen.aspx

[34] International Journal of Health Management and Tourism » Submission » MEDICAL TOURISM COMPETITION: THE CASE OF TURKEY [Internet]. [cited 2024 Mar 3]. https://dergipark.org.tr/en/pub/ijhmt/issue/36742/372364

[35] World Population Review [Internet]. [cited 2024 Mar 3]. https://worldpopulationreview.com/country-rankings/immigration-by-country%202022

[36] TheGlobalEconomy.com [Internet]. [cited 2024 Mar 3]. Female labor force participation by country, around the world. https://www.theglobaleconomy.com/rankings/female_labor_force_participation/

[37] Dungan D, Qualtrics. 2023 [cited 2024 Mar 3]. Countries Ranked by Percentage of Workforce Who Are Women. https://www.qualtrics.com/blog/countries-ranked-by-female-workforce/

[38] List of countries. by traffic-related death rate. In: Wikipedia [Internet]. 2024 [cited 2024 Mar 3]. https://en.wikipedia.org/w/index.php?title=List_of_countries_by_traffic-related_death_rate&oldid=1211581529

[39] Jordan Times [Internet]. 2021 [cited 2024 Mar 3]. Cost of road accidents in Jordan decreased in 2020 — report. https://jordantimes.com/news/local/cost-road-accidents-jordan-decreased-2020-%E2%80%94%C2%A0report

[40] TobaccoAtlas_6thEdition_LoRes.pdf [Internet]. [cited 2024 Mar 3]. https://theunion.org/sites/default/files/2020-12/TobaccoAtlas_6thEdition_LoRes.pdf

[41] Breaking the habit. Tobacco control & behavioral solutions can save lives in Jordan [Internet]. 2023 [cited 2024 Mar 3]. https://blogs.worldbank.org/arabvoices/breaking-habit-tobacco-control-behavioral-solutions-can-save-lives-jordan

[42] Musaiger AO, Al-Hazzaa HM, Takruri HR, Mokhatar N. Change in nutrition and lifestyle in the eastern mediterranean region: health impact. J Nutr Metab. 2012;2012:436762. 22720142 10.1155/2012/436762 PMC3375092

[43] Rahim HFA, Sibai A, Khader Y, Hwalla N, Fadhil I, Alsiyabi H, et al. Non-communicable diseases in the arab world. Lancet Lond Engl. 2014;383(9914):356–67. 10.1016/S0140-6736(13)62383-1 24452044

[44] Chooi YC, Ding C, Magkos F. The epidemiology of obesity. Metabolism. 2019;92:6–10. 30253139 10.1016/j.metabol.2018.09.005

[45] Jordan News | Latest News from Jordan, MENA [Internet]. 2022 [cited 2024 Mar 3]. Obesity costing Jordan JD650 million annually - Jordan News | Latest News from Jordan, MENA. https://www.jordannews.jo/Section-109/News/Obesity-costing-Jordan-JD650-million-annually-17144

[46] TheGlobalEconomy.com [Internet]. [cited 2024 Mar 3]. Hospital beds per 1,000 people by country, around the world. https://www.theglobaleconomy.com/rankings/hospital_beds_per_1000_people/

[47] List of countries. by hospital beds. In: Wikipedia [Internet]. 2024 [cited 2024 Mar 3]. https://en.wikipedia.org/w/index.php?title=List_of_countries_by_hospital_beds&oldid=1210184825

[48] Gericke CA, Britain K, Elmahdawy M, Elsisi G. Health System in Egypt. In: van Ginneken E, Busse R, editors. Health Care Systems and Policies [Internet]. New York, NY: Springer US; 2018 [cited 2024 Mar 3]. pp. 1–19. (Health Services Research). 10.1007/978-1-4614-6419-8_7-2

[49] Statista [Internet]. [cited 2024 Mar 3]. Turkey: population covered by health insurance 2021. https://www.statista.com/statistics/1272717/population-covered-by-public-or-private-health-insurance-in-turkey/

[50] Bernal-Delgado E, Garcia-Armesto S, Oliva J, Sanchez Martinez FI, Repullo JR, Pena-Longobardo LM, et al. Spain: Health Syst Rev Health Syst Transit. 2018;20(2):1–179. 30277216

[51] Statista [Internet]. [cited 2024 Mar 3]. Healthcare spending as a percentage of GDP by country 2022. https://www.statista.com/statistics/268826/health-expenditure-as-gdp-percentage-in-oecd-countries/

